# Dr. Alder's Proofs of His Theory of the Proximate Causes of Diseases from Anatomy. In Continuation of His Letters on Fevers, Contagion, and Quaratines

**Published:** 1809-04

**Authors:** Thomas Alder


### Dr. Alder's *Proofs of his Theory of the proximate Causes of Diseases from Anatomy. In Continuation of his Letters on Fevers, Contagion, and Quaratines.*

It appears to me, it would be far from improper for me here to animadvert upon the opinions of Dr. Hendy, Dr. Beddoes, Dr. Clarke, some writers in the New York Medical Repository, and others, who have attributed diseases to too much oxygen in the air; and in particular, upon some eudiometric experiments.

I have some little additions to make to the matter of my last: the venous congestions which I spoke of, do not always consist *entirely of blood;* they often have much air mixed with them, and sometimes are composed chiefly *of air,* as I shall soon proceed to show by dissections. We might in some measure have anticipated this, as the intestinal canal is always considerably disordered in these complaints; much air is generated in it, and air will be absorbed from it by the lacteals as well as chyle. The symptoms too of several of these diseases, and in many instances of almost all of them, are attributable to congested *gas* rather than altogether to congested blood. For example, in hysteria, hypochondriasis, and those other disorders which have been called *nervous,* the congestions often quickly remove from one place to another, and sometimes so as to mimmic a variety of diseases. The same happened in a great part of those intermitting fevers which Cleghorn treated at Minorca. In considering congestive diseases, therefore, we should always keep in view that congestions *may be* formed *chiefly of gas.* This being added to my Theory, accounts (as I judge, in a satisfactory manner) for the sudden attack and shifting of many symptoms symptoms, as well as in some measure for the success of several stimulating remedies; and is in part proved by these circumstances, though much more clearly by the proofs of the formation of gas in the intestines, and of its absorption from them, and by such gas being found congested in those complaints, on dissection.

I come now to the *anatomical proofs of the preceding Theory.*

In examining dead bodies, in order to discover the proximate causes of diseases, various circumstances are to be taken into consideration.

1st. We sometimes can find very little more by dissections than the RESULTS of the disease; and this has made so great an impression upon the very learned Professor of the Practice of Physic at Edinburgh, that he, in appearance chiefly on this account, has given up all hopes of any thing important being discovered, in most diseases, from pathological anatomy. But with deference to this great character, I am clearly of opinion we may trace *effects* back to their *causes,* in the morbid appearances that take place in almost every disease.

2dly. The Dissectors tell us, in their examinations of the bodies which have died from some of the worst diseases, that *they have often found nothing morbid;* and this has made a greater impression upon them, and indeed, I believe I may say, upon all medical men, than the preceding; and has led them to believe, that the principal injury to the system, in such diseases, has been in the *nerves,* the disorders of which appear in general to be *inscrutable.* But this objection against pathological anatomy, and difficulty in it, is no more to be dwelled upon than the former. For Dissectors, in such cases, do not use *correct terms.* I never saw *any* body opened, *where the appearances were not morbid!* Morbid circumstances take place *in articulo mortis.* The lungs are congested; the veins are filled; and the arteries are emptied. In consequence of this, the veins in the brain and in other parts, which are the soonest overcharged by an impeded passage of the blood through the lungs, are more or less congested *in almost every dead body,* as well as the lungs; and sometimes very considerably when the patient has had no signs of any disease in those parts while living! The dissectors therefore, ought to say they find nothing *unusual* when they tell us they find nothing morbid. So far with respect to *this part* of this objection and difficulty. But another difficulty in and objection against pathological anatomy, it is is evident, arises out of the explanation which I have given: for, if so many morbid circumstances take place in almost all cases of death, and while a person is dying, and these sometimes are so considerable, how (it may rationally be asked) are we to distinguish such circumstances as take place *during death,* from such as *existed in and constituted the previous disease?* The answer, however, to this question, (notwithstanding it plausibility) is not very difficult; in by far the greater number of cases those congestions spoken of are much greater when they have formed *the disease,* than when they have come on while a person has been dying; and, in a very great number of cases, there are other proofs that they constituted the disease, more strongly than this;—for example, the symptoms *of the disease* indicate such congestions; and these congestions are extremely often found to have run on to great effusions or extravasations; to inflammation or gangrene; to any of which states they would scarcely ever have time to run during death—during death from a cause I mean no way connected with such congestions. But during death, when a person dies from such congestions, as in an apoplectic or epileptic fit, &c. effusions or extravasations may take place, and the congested parts may appear as if inflamed.

3dly. Polypi often form in the veins *during diseases,* and greatly alter the phænomena.

4thly. Parts change *after death.* Thus, if a dead body be tumbled about, the blood will run from one part into another. And serous membranes, when congested with red blood, soon exclude it. Congested air too may escape. And the stomach may be eroded by its own juice. I need scarcely add also, partial putrefaction will sometimes suddenly come on; and, besides altering parts, it may generate a large quantity of air. Polypi often form too during death.

In pathological dissections, these circumstances certainly ought constantly to be kept in view. But when they are so, this branch of medicine will not be found so perplexing by far, as it has been represented to be, nor nearly so uninstructive. And I persuade myself, it speaks sufficiently plain to light me clearly in my way through the comprehensive and momentous subjects in which I am now engaged. For instance, with these in view, I am not at all embarrassed, when I confess, that after apoplexy or epilepsy, sometimes nothing has been found peculiar, (or as it is usually termed, "*morbid*") either in the brain or in any any other part; and the same may be said of fever and of some of the other complaints, of the proximate causes of which I am now treating. For this is clearly owing to congestions uniformly taking place in these organs, especially in their veins, or in some degree *in almost all cases of death.* And in general, I know from many great authorities that these congestions are rather peculiar, and have often run on to further consequences, as effusion or extravasation, &c. I suppose I have read the accounts of near one hundred dissections after apoplexy, and of near fifty after epilepsy, and of about two hundred or more after fevers, of all the different kinds. For apoplexy my chief authors were Wepferus and Morgagni; for epilepsy, Morgagni and numerous others, who had detailed single cases, or collected the *results* of many. For fever, the authors of the *Traitè des Causes, &c. de la Peste* (who detail about twenty-five cases which they had dissected themselves, and relate the appearances which were observed in about twenty-five more, in sailors, who were dissected at Brest); and our own practical writers, as Cleghorn, Lind, Jackson, Chisholm and Pringle, (who relate about twenty-five); also the late Americans, as Rush, Rand, and Warren, Mr. Ffirth, &c.; Assalini too, and Hoffman, Senac, and Morgagni, with perhaps a few others whom I cannot now recollect. As for disorders of the head, certainly after apoplexy and epilepsy, &c. organic diseases of all kinds have often, in different cases, been found in every part of the brain, and several of these no doubt were the original causes of these diseases; but many of them, on the other hand, it is most probable, took place from habitual congestions in the head, occasioned either by the disease itself, or by the proximate cause of it.

But whether these chronic organic affections existed or not, *in by far the greatest number of cases,* the congestions in many parts of the brain were very great, so as frequently to make the dissectors say, certain parts of the brain were *inflamed*; and these have commonly gone on to both extravasation of red blood and effusion of the colourless part of it, but sometimes only to one of them, and in those of a languid circulation, perhaps, only to effusion. The appearances after apoplexy and epilepsy are very similar; but after epilepsy there is perhaps oftener some irritating projecting substance pressing upon the brain (as bone), and after apoplexy there probably oftener is great sanguine extravasation.

Yet after the very violent spasmodic (often tetanic) af-

 fections fections which carried off numbers of newly-landed soldiers in the East-Indies, just when the suffocating land winds had given place to the cold damp atmosphere of the monsoons, (as related by Dr. Girdlestone in Dr. Clarke's work) on dissection, " it appeared that no injury had been done to the brain, liver, gall-bladder, stomach, or heart, whether the patients died of universal spasms or an apoplexy." These are rather uncommon cases; it appears from the symptoms, that cold and moisture checked the *whole circulation*, so that the blood was not carried very fast up into the large veins; and as the patients died quickly, it, *at the rate it went*, often had scarcely time to form large congestions. But another important circumstance is, the patient's body was very cold, and this would constringe the veins so as to make them *resist* congestions. The lungs even were cold, so that " the breath was so condensed as to be both seen and felt, issuing in a cold stream, at a considerable distance." But after all, it is pretty certain that the organs mentioned, as well as the lungs, were congested, *as they usually are in articulo mortis*, and therefore were injured so as to occasion the disease and death. I do not find that the state of the lungs was noticed.

I apprehend this complaint was very similar to the tetanus from cold, which so often attacks children and negroes in the West Indies; but much more severe. But as death from concentrated bad air, very frequently or mostly is a sanguineous apoplexy, so the congestions in the lungs and head, as found uniformly in five or six dissections made by Portal, as well as in those made by others, after this, (the disease being apoplexy) are as considerable as those which usually take place in common apoplexy; and this circumstance, together with the *very cold breath*, mentioned in the last cases, of themselves prove that all these disorders arise from an impeded passage of the blood through the lungs. As death too, from suffocation in any other way, is also pretty much of the same kind, so the appearances presented by dissection are also much the same. But some variation will occur from the body being different in temperature in different cases, while it is suffocating, and also from some other circumstances.

I am more full upon these impeded pulmonary transmissions which more especially affect the head than I otherwise should be, because they are the most common, and the most alarming among the symptoms of fever. But it must must be added, that in these cases, where *the head* is the most materially injured by venous congestions, occasioned by an obstructed pulmonary transmission, that the veins in general, and the other organs which most materially and quickly empty their blood into them, are all more or less congested at the same time. Thus, in the dissections after apoplexy (in particular) of Wepfer, Morgagni, Portal, and others, where other parts than the head are noticed, large congestions in the lungs, in the right side of the heart, and in the venæ cavæ, in the liver, and sometimes in the intestines, are mentioned as the common appearances; and from these, as well as from a knowledge of the nature of the proximate causes of such complaints, and also sometimes from symptoms which occur in them, we have every reason to conclude, that if the proximate cause of apoplexy, and those other diseases which attack principally the brain, did not prove so suddenly fatal by attacking the head, it would cause fevers, even with diseases in the liver or intestinal canal, or both, and particularly if any thing noxious acted especially on these organs. In the dissections of people who had died from an impeded pulmonary transmission, congesting principally the liver, or intestines, or both; in the first instance, producing hepatitis; in the last, commonly dysentery; and in the other cholera with fever; we seldom fail to find too, that the head (on the other hand) had been congested in some degree whenever it had been accurately examined. But as these complaints when so joined are modifications of fever much more strictly than apoplexy or epilepsy can in general be said to be so; and as dissectors have not, in these, much attended to any but what they conceived the *immediate seats* of these diseases, I shall specify the appearances found left by them in the dead body when I come to fevers; noticing only that Sir John Pringle's cases of dysentery were merely subsequent to fever, and usually long preceded by a diarrhœa; both which circumstances caused venous congestions to be partial.

But the venous congestions which are found after these diseases are not always entirely *of blood*; Morgagni says, in Letter V. article 19, "A fisherman afflicted with a rupture, being liable to flatulent complaints in his belly, was seized with them as he sat in his boat, and immediately died. I dissected his body the day after death. His abdomen was swelled from the stomach and intestines being distended with air. The gastro-epiploic vein was so tumid that it would every where *admit my fore-finger*, yet no no sooner was it cut into than it shrunk, for it contained a large quantity of air. The heart was large and flaccid, and black *frothy* blood was found in *both* its ventricles, and in the right auricle; *nor was there any vein through the whole body wherever we dissected which was not distended with frothy black blood. A little* also of the same was seen in the aorta and carotids, and the trunk of the pulmonary was turgid with this kind of blood, joined also with air. In the cranium the sinusses, but particularly the vessels which go through the dura mater, were turgid with frothy blood; as were all the vessels which run through the pia mater, whether in the basis or circumference of the cerebrum and cerebellum, or in the ventricles. And the smaller vessels were still more turgid than the larger ones, &c." Morgagni goes on, relating many other cases where apoplexy or epilepsy had been caused by congested air absorbed from the intestines; and mentions some too where air had been taken into the vessels from impeded respiration. Portal mentions many of this latter kind; and Lieutaud relates one of the former kind, and says, Willis had seen many of them.

My conclusion then, from all these cases is,—the veins and the organs more largely emptying themselves into them, are always congested in apoplexy, and probably always in epilepsy, except when this arises from evacuations; and generally chiefly with blood, though sometimes in a great measure from air; notwithstanding that great organic affections often excite the disease, and notwithstanding too, that these congestions, in several cases, are not greater than such as in common cases are formed *in articulo mortis*.

The *necessity* for my taking up so much time in proving venous and cephalic congestion in these diseases may be justly lamented; but as the labour of it must be all dead fag to me, I hope it will be attributed to its proper motive. In my next I mean to go on to the anatomical proofs of the proximate cause of fever.

I am, &c.

THOMAS ALDER, M. D.

*Additional*

